# Advanced bioactive nanomaterials for diagnosis and treatment of major chronic diseases

**DOI:** 10.3389/fmolb.2023.1121429

**Published:** 2023-01-26

**Authors:** Yongfei Liu, Yi Yi, Chengqian Zhong, Zecong Ma, Haifeng Wang, Xingmo Dong, Feng Yu, Jing Li, Qinqi Chen, Chaolu Lin, Xiaohong Li

**Affiliations:** ^1^ Department of Urology, Longyan First Hospital Affiliated to Fujian Medical University, Longyan, China; ^2^ Longyan First Hospital Affiliated to Fujian Medical University, Longyan, China

**Keywords:** nanomaterials (A), neurodegenerative disease, cancer, bioactivity, biomimetic nanomaterials, inorganic nanomaterials, organic nanomaterials, nanozyme

## Abstract

With the rapid innovation of nanoscience and technology, nanomaterials have also been deeply applied in the medical and health industry and become one of the innovative methods to treat many diseases. In recent years, bioactive nanomaterials have attracted extensive attention and have made some progress in the treatment of some major chronic diseases, such as nervous system diseases and various malignant tumors. Bioactive nanomaterials depend on their physical and chemical properties (crystal structure, surface charge, surface functional groups, morphology, and size, etc.) and direct produce biological activity and play to the role of the treatment of diseases, compared with the traditional nanometer pharmaceutical preparations, biological active nano materials don’t exert effects through drug release, way more directly, also is expected to be more effective for the treatment of diseases. However, further studies are needed in the evaluation of biological effects, fate *in vivo*, structure-activity relationship and clinical transformation of bionanomaterials. Based on the latest research reports, this paper reviews the application of bioactive nanomaterials in the diagnosis and treatment of major chronic diseases and analyzes the technical challenges and key scientific issues faced by bioactive nanomaterials in the diagnosis and treatment of diseases, to provide suggestions for the future development of this field.

## 1 Introduction

Nanomaterials are short for nanoscale structural materials. In a narrow sense, they refer to solid materials composed of nanoparticles with a size of no more than 100 nm, and in a broad sense, they refer to all kinds of solid ultra-fine materials with at least one dimension of the three-dimensional spatial scale of microstructure in the nanometer scale (1–100 nm) (Cheng et al., 2020). Nanotechnology is a comprehensive subject with strong intersection, and the research content involves a broad field of modern science and technology, from microtechnology including microelectronics to nanotechnology. Human beings are becoming more and more in-depth to the microscopic world, and the level of people’s understanding and transformation of the microscopic world has increased to an unprecedented height. At present, nanotechnology has included nano-electronics, nano-mechanics, nanomaterials science, nano-chemistry, nano-biology, and other disciplines. With the continuous research and development of nanoscience and technology, it has been widely used in energy and environment, electronics and information, medicine, and health (Zheng et al., 2015; Li et al., 2017a; Kumawat et al., 2017), etc., and has a profound impact on the rapid development of related industries. Biological detection, drug delivery, and disease diagnosis and prevention have become the research hotspots of nanoscience in the field of health care. Currently, ph-responsive, and enzyme-responsive nanomaterials, which are widely used in targeted drug delivery and controlled drug release (Zhao et al., 2021), are materials that can change their physical and chemical properties (such as surface charge and chemical structure) in response to external stimuli such as light and heat, reactive oxygen species (ROS) levels, and pH changes. In recent years, bioactive nanomaterials have attracted extensive attention and attention. Bioactive nanomaterials are bioactive nanomaterials that interact with proteins, cells or tissues *in vivo* and cause biological reactions depending on their physical and chemical properties (crystal structure, surface charge, surface functional group, morphology and size, etc.) (Zhou et al., 2019a; Xu et al., 2021). Since Larry Hench in the 60 s of last century Since the concept of bioactive materials was first proposed in the 1960s by discovering that bioactive glass can closely integrate with the surrounding bone tissue at the interface (Henchll, 2002), bioactive nanomaterials have been developed with nanoscale size and precise structure, which enable them to accurately regulate the interaction between materials and biological systems, and thus exhibit unique biological activities (Islam et al., 2020). This is also far beyond the scope defined by Larry Hench in the past. Due to the absence of therapeutic drug loading and drug release process, bioactive nanomaterials are more direct than the target mode of action, which is expected to achieve better therapeutic effects, and have made certain research progress in the treatment of some major chronic diseases such as nervous system diseases and various malignant tumors. However, there are still few systematic summaries of bioactive nanomaterials and their related applications. This article reviews the latest research progress and reports of bioactive nanomaterials in the diagnosis and treatment of major chronic diseases, systematically introduces the typical applications of bioactive nanomaterials in the biomedical field and analyzes the technical challenges and key scientific issues faced by bioactive nanomaterials in the diagnosis and treatment of diseases, to provide suggestions for the future development of this field.

## 2 Influencing factors of biological activity of nanomaterials

Traditional biological nanomaterials respond to external stimuli such as pH changes, reactive oxygen species levels, light and heat, and then change their physical and chemical properties such as surface charge and chemical structure to play a role. However, the chemical structure and surface properties of bioactive nanomaterials are usually relatively clear (Kerativitayanan et al., 2015), and they directly interact with proteins, cells, or tissues *in vivo* and cause biological reactions through their physical structure, surface properties and nano-topography (Zhou et al., 2019b). Therefore, these characteristics such as structure and properties are important factors affecting the biological activity of materials.

### 2.1 Nano-morphology

Studies have found that the adhesion of Embryonic stem cells (ESCs) is affected by the roughness of the surface (Chen et al., 2012). Compared with the rough surface, the smooth surface is easier to make undifferentiated cells adhere. In addition, rough surfaces can induce the differentiation of ESCs, while smooth surfaces can maintain the self-renewal ability of ESCs. Kwon et al. also showed that cells on surfaces with different roughness or different nanotopography would exhibit distinct behaviors (Kwon et al., 2012), and these studies revealed that cell behaviors were affected by nanotopography.

### 2.2 Surface properties

The biological activity of bioactive nanomaterials is affected by surface properties. For example, bioactive ligands such as small molecules, peptides and proteins are modified to the surface of materials by chemical modification (Eivazzadeh-Keihan et al., 2020), and the surface charge, hydrophilic and hydrophobic properties of bioactive nanomaterials are adjusted to become bioactive nanomaterials with specific biological functions. Some studies have found that the use of polymer nanoparticles to modify the surface of cellular-mesenchymal epithelial transition factor (c-MET) peptide bioactive nanoinhibitors and Mesenchymal epithelial transition factor (MET). The affinity of MET is three orders of magnitude higher than that of free c-MET peptide (KD = 3.96 × 10-7 mol/L) (KD = 1.32 × 10-10 mol/L) (Wu et al., 2018). It has also been found that positively charged (+7 mV) Au NPs have no effect on the aggregation of Aβ protein, while negatively charged (−38 mV) Au NPs can effectively inhibit the aggregation of β-amyloid (Aβ) to form toxic oligomers.

### 2.3 Physical structure

The physical structure of nanomaterials can affect their biological activity. For example, Molecular imprinting polymer (MINP) can bind target biomolecules with high affinity. The specific biological activity depends on the fine structure of the nanoparticle itself. Different fine structures show different biological activities. Some scholars have developed a MINP that can capture vascular epidermal growth factor and thus reduce angiogenesis in the tumor to inhibit tumor growth (Koide et al., 2017), and some studies have reported a borate-based MINP that inhibits tumor growth by blocking the human epidermal growth factor receptor-2 signaling pathway (Dong et al., 2019). These studies have shown that MINP with different fine structures can be developed for the treatment of various diseases such as cancer (Tang et al., 2017; Zhang, 2020). In addition, the particle size of the material also plays an important role in the influence of its biological activity. The specific surface area of nanoparticles is opposite to the particle size, the smaller the particle size, the larger the specific surface area. Jong et al. (Park et al., 2011) found that Ag NPs and Ag+ with large particle size were less toxic by measuring the cytotoxicity of Ag NPs, which are widely used in medicine for antibacterial treatment. Therefore, particle size plays an important role in determining the biological activity of nanomaterials.

## 3 Classification of bioactive nanomaterials

With the rapid development of materials science and the development of various bioactive nanomaterials, bioactive nanomaterials have been widely used in biomedicine. Bioactive nanomaterials can be classified into organic nanomaterials, inorganic nanomaterials, bioactive nanoenzymes, and biologically active biomimetic nanomaterials.

### 3.1 Bioactive organic nanomaterials

**FIGURE 1:**
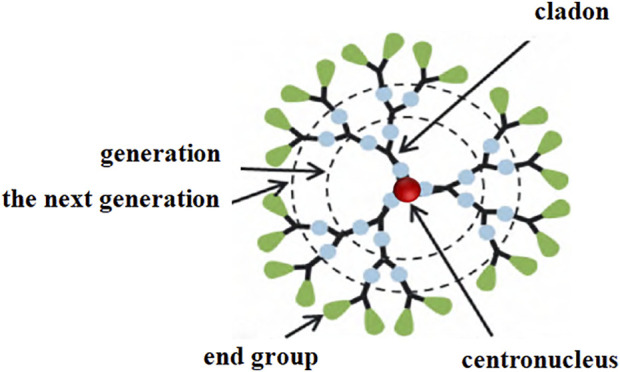
Schematic of the basic structure of the tree molecule.

Bioactive organic nanomaterials include bioactive nanofibers and bioactive tree molecules. Nanofibers have the characteristics of high specific surface area, high porosity, and good mechanical properties (Shikhi-Abadi and Irani, 2021). One-dimensional nanolinear assemblies with a diameter of 50–500 nm and an aspect ratio of more than 1: 200 are prepared from organic polymer solutions or melts, which have antibacterial and anti-inflammatory properties. For example, poly (ε -caprolactam)—β -poly (ethylenimine) (PCL- β -PEI) nanofibers can prevent CpG oligodeoxynucleotide (ODN) from stimulating dendritic cells and macrophages to secrete cytokines α, tumor necrosis factor (TNF-α) and interferon-γ by electrostatic adsorption of ODN (Kang and Yoo, 2014). While N-trimethyl chitosan nanofibers can generate pressure by electrostatic binding of polycations on the membrane to negatively charged parts of the bacterial cell wall, leading to lysis and death of bacterial cells, and then inhibit inflammatory response (Cheah et al., 2019). However, such nanofibers are prepared based on cationic polymers, and the cell membrane of mammals is also negatively charged, so it is easy to produce cytotoxicity. Tree molecules are usually a kind of spherical nanoscale molecules (Figure 1) composed of three parts: a central core, a branching unit, and a terminal group. The more generations they have, the larger the particle size. Tree molecules can inhibit virus entry into host cells by modifying groups that block the ability of virus to attach to host cells, modifying cationic groups such as zwitterions (Mintzer et al., 2012), organic metals (Abd-El-Aziz et al., 2015), amino acids, and glycopeptides (Michaud et al., 2016), etc. The introduction of hydrophobic chains can damage the cell membrane (Zielińska et al., 2015) or enhance the electrostatic interaction with the bacterial cell membrane to play a role in anti-infection and anti-inflammation. Polyamide amine tree molecules with carboxyl and benzene ends can inhibit the aggregation of β-amyloid peptides through hydrophobic binding and electrostatic repulsion, thus playing a role in nervous system diseases (Wang et al., 2018; Wang et al., 2019a). Polyacylthiourea tree molecules can be modified by polyethylene glycol (PEG) to efficiently chelate copper ions, thereby downregulating the expression of vascular endothelial growth factor (VEGF) in tumor sites and inhibiting the formation of tumor neovascularization (Shao et al., 2017), thus achieving anti-tumor effects. However, few bioactive tree molecules have entered clinical research and application, and only one naphthalene disulfonate-modified polylysine tree molecule has been approved as an antiviral additive for condoms in Australia, which needs to be further evaluated for safety and biocompatibility.

### 3.2 Bioactive inorganic nanomaterials

Inorganic nanomaterials are a class of nanomaterials with inorganics as the main body (Nethi et al., 2019), including biologically active carbon nanomaterials, biologically active precious metal nanomaterials, biologically active metal oxide nanomaterials, and biologically active non-metallic nanomaterials. They usually have higher mechanical stability.

#### 3.2.1 Bioactive noble metal nanomaterials

Nanomaterials prepared from gold, silver, platinum, etc., are commonly referred to as precious metal nanomaterials. It has been found that gold nanomaterials can exert antibacterial, anti-inflammatory, anti-tumor, and other biological activities according to their specific size, morphology and surface sealing groups. Gold nanoparticles with a size of about 1 nm can induce the production of many reactive oxygen species (ROS) in bacteria, and gold nanopins with high aspect ratio can induce bacterial dissolution through mechanical pressure to achieve the purpose of antibacterial (Zheng et al., 2017; Elbourne et al., 2019). It can also achieve anti-inflammatory effect by regulating related signaling pathways. Some studies have found that gold nanomaterials also have a certain effect on anti-tumor (De Carvalho et al., 2018; Hao et al., 2021), but the structure-activity relationship and biological effect need to be further studied. In particular, the potential toxicity caused by long-term accumulation of inorganic materials in organs and tissues needs to be further clarified.

#### 3.2.2 Biologically active non-metallic nanomaterials

Common bioactive non-metallic nanomaterials mainly include selenium and black phosphorus. As one of the essential non-metallic elements for human body, selenium exerts its biological activity by binding to the structure of selenoproteins in the body. Selenium nanoparticles can exert anti-inflammatory and anti-oxidative effects by activating Nrf2 and its downstream genes, inhibiting ROS production and scavenging superoxide and DPPH free radicals (Cheng et al., 2017; Song et al., 2017). It can also achieve antibacterial effects by inducing ROS production, consuming internal ATP to interfere with bacterial metabolism, destroying membrane structure, disturbing membrane potential, and decomposing mature extracellular polysaccharide matrix produced by bacteria (Cremonini et al., 2016; Huang et al., 2019). Black phosphorus (BP) nanosheets can induce bacterial apoptosis by producing ROS, and can cause physical damage to the bacterial cell membrane to kill bacteria to achieve antibacterial effect (Xiong et al., 2018). At the same time, it can also interfere with cell multipolar spindle and mitosis, and cause cell apoptosis, thereby exerting great anti-tumor potential (Shao et al., 2021).

#### 3.2.3 Biologically active carbon nanomaterials

**FIGURE 2:**
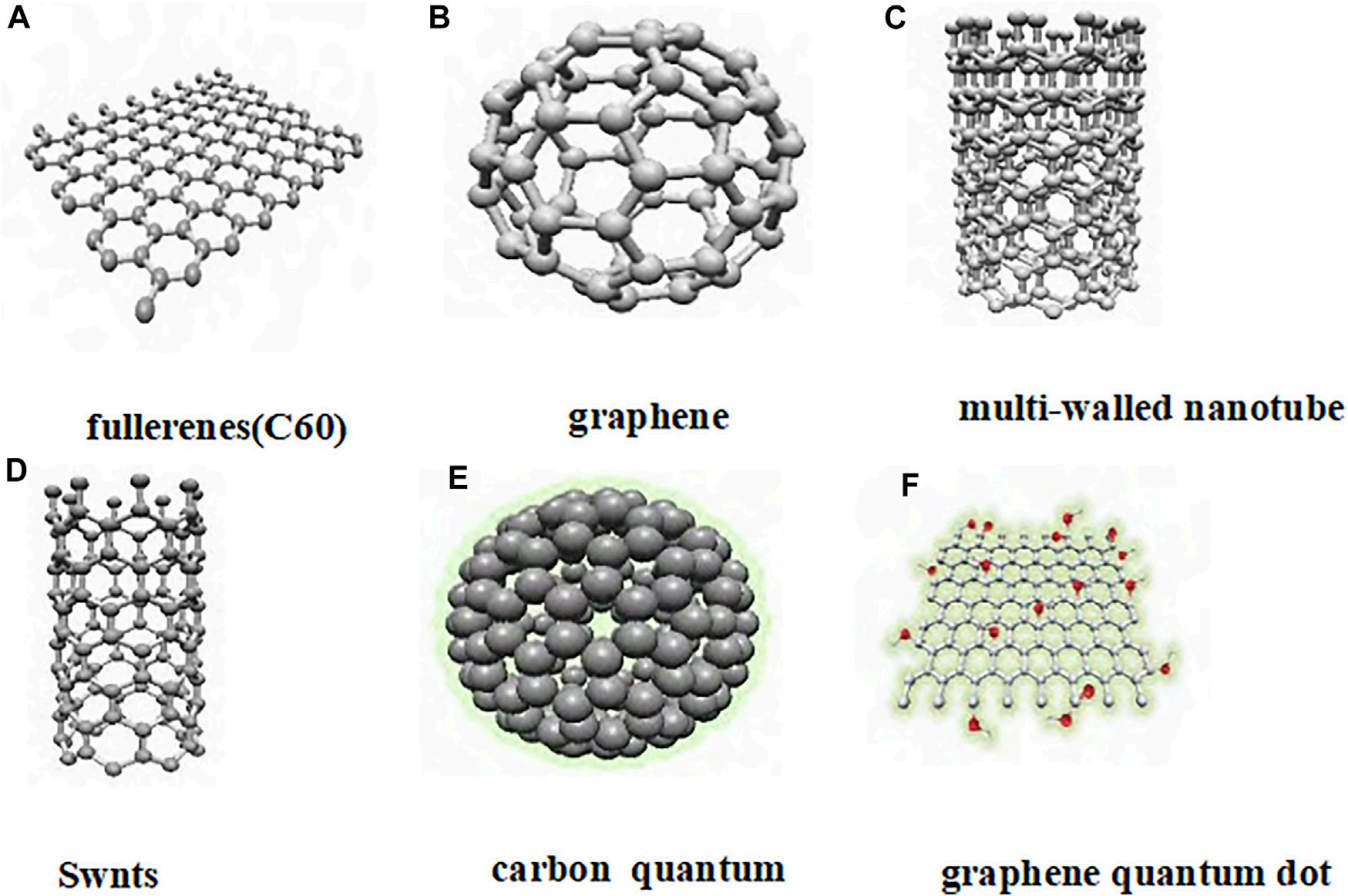
Different types of carbon nanomaterials.

Carbon nanomaterials have attracted increasing attention due to their unique electrical, optical, thermal, and mechanical properties (Wang et al., 2014; Lin et al., 2016; Tiwari et al., 2016; Zhang et al., 2017) Carbon nanomaterials include graphene, fullerene (C60), carbon nanotubes, carbon dots, graphene quantum dots, etc., (Figure 2). Carbon nanomaterials are widely used in the biomedical field due to their excellent biological activity and controllable functional design. Studies have found that carbon nanomaterials not only have nanoenzyme activity, but also have antibacterial, anti-infection, anti-tumor, and other biological activities. For example, graphene oxide (GO) nanosheets can affect the formation of dendritic cell -T cell synapses and enhance the activation and proliferation of antigen-specific CD8^+^ T cells as DC vaccine adjuvants, thus playing an anti-infection role (Zhou et al., 2021). Quaternary ammonium modified carbon quantum dots (QCQD) can play an antibacterial role by interfering with protein translation, post-translational modification, and protein transport in bacteria (Zhao et al., 2020). Graphy-oxide acetylic acid (GDYO) can interact with signal transduction proteins and transcription factor STAT3 in the intracellular, and turn the pro-tumor M2 macrophages into anti-tumor M1 macrophages, thereby reversing the tumor immunosuppressive microenvironment and playing an anti-tumor role (Guo et al., 2021a). However, there are some key problems in the cli nical transformation of carbon nanomaterials, such as the metabolism and clearance process in the body cannot be fully elucidated, and their safety *in vivo* needs to be further studied.

#### 3.2.4 Bioactive metal oxide nanomaterials

Metal oxide refers to the binary compounds composed of oxygen and another metal chemical element, including basic oxide, acid oxide, peroxide, superoxide, amphotericin oxide, etc. In addition to the high specific surface area and high mechanical strength due to its size effect, metal oxide nanoparticles also have the advantages of wide source and stable structure. They play a very important role in many fields such as physics, chemistry, and materials science. It can exhibit insulator, semiconductor or metal characteristics depending on the oxidation state of the metal and the environment. Studies have confirmed that metal oxides of specific sizes have anti-inflammatory, antibacterial, anti-tumor, and other biological activities. For example, tiO2 nanoparticles can inactivate thrombin by promoting the formation of thrombin-antithrombin complex in plasma, thereby blocking the way that thrombin causes inflammatory response through protease activated receptors, and can inhibit oxidative stress response induced by the activation of Toll-like receptors on the surface of platelets (Seisenbaeva et al., 2017). In addition, zno nanoparticles can also improve the antioxidant capacity of the colon and reduce inflammatory damage (Li et al., 2017b). Nanoparticles such as zinc oxide, copper oxide and iron oxide can achieve anti-tumor effects by causing membrane leakage of tumor cells, inducing oxidative stress and promoting apoptosis (Wahab et al., 2014; Nagajyothi et al., 2017; Yousefvand et al., 2021).

### 3.3 Bioactive nanozymes

Nanomaterials containing catalytic properties like those of natural enzymes are called nanozymes (Wei and Wang, 2013; Wu et al., 2019), including multiple enzyme-like active nanozymes, peroxidase-like and oxidases, catalase-like active nanozymes, superoxide dismutase-like active nanozymes, etc., due to their low cost, good stability, and easy mass production. They are widely used in many fields such as biomedicine, physical chemistry, materials, agriculture, environmental management, national defense, and security (Wang et al., 2016). A variety of enzyme-active nanozymes can exhibit different types of enzyme-like activities under different conditions. For example, manganese dioxide doped nanoparticles (MnO2 NPs) have a variety of enzyme-like activities that are more stable than natural enzymes, and nitrogen carbon nanomaterials (N-CNMs) can simulate a variety of enzyme-like activities, which can change the intracellular microenvironment of tumor cells and achieve anti-tumor effects (Fan et al., 2018).

### 3.4 Biologically active biomimetic nanomaterials

By learning the micro and nano multi-scale structure, composition, function and principle in life systems, nanomaterials are designed and prepared to imitate various functions in life systems, which are called biomimetic nanomaterials. These materials include bioactive biomolecular assembly nanomaterials, bioactive cell-like nanomaterials, etc. Biomacromolecules in the body can achieve similar biological activities by rationally designing the physical and chemical properties of materials and mimicking the structure and properties of natural biomacromolecule assemblies. For example, the synthesis of quinazolinone derivatives with arylboric acid connecting groups (BQA-GGFF) can simulate the neutrophil extracellular traps to capture pathogenic microorganisms *in vivo*, thus playing an antibacterial role (Huang et al., 2020). Polymer micelles prepared by polyuamine- β -polycaprolactone (PAE- β -PCL) and polyethylene glycol- β -polycaprolactone (PEG-β-PCL) mimic the role of heat shock protein molecular chaperones to specifically recognize and adsorb hydrophobic fragments in abnormal proteins, so as to play a therapeutic effect in inflammatory response and nervous system diseases (Xu et al., 2019).

## 4 Application of bioactive nanomaterials in biomedicine

After decades of development, bioactive nanomaterials have been widely used in real life. They have been well used in anti- infection therapy, inflammatory disease treatment, cancer treatment, and neurodegenerative disease treatment.

### 4.1 Application of bioactive nanomaterials in anti-infection

**FIGURE 3:**
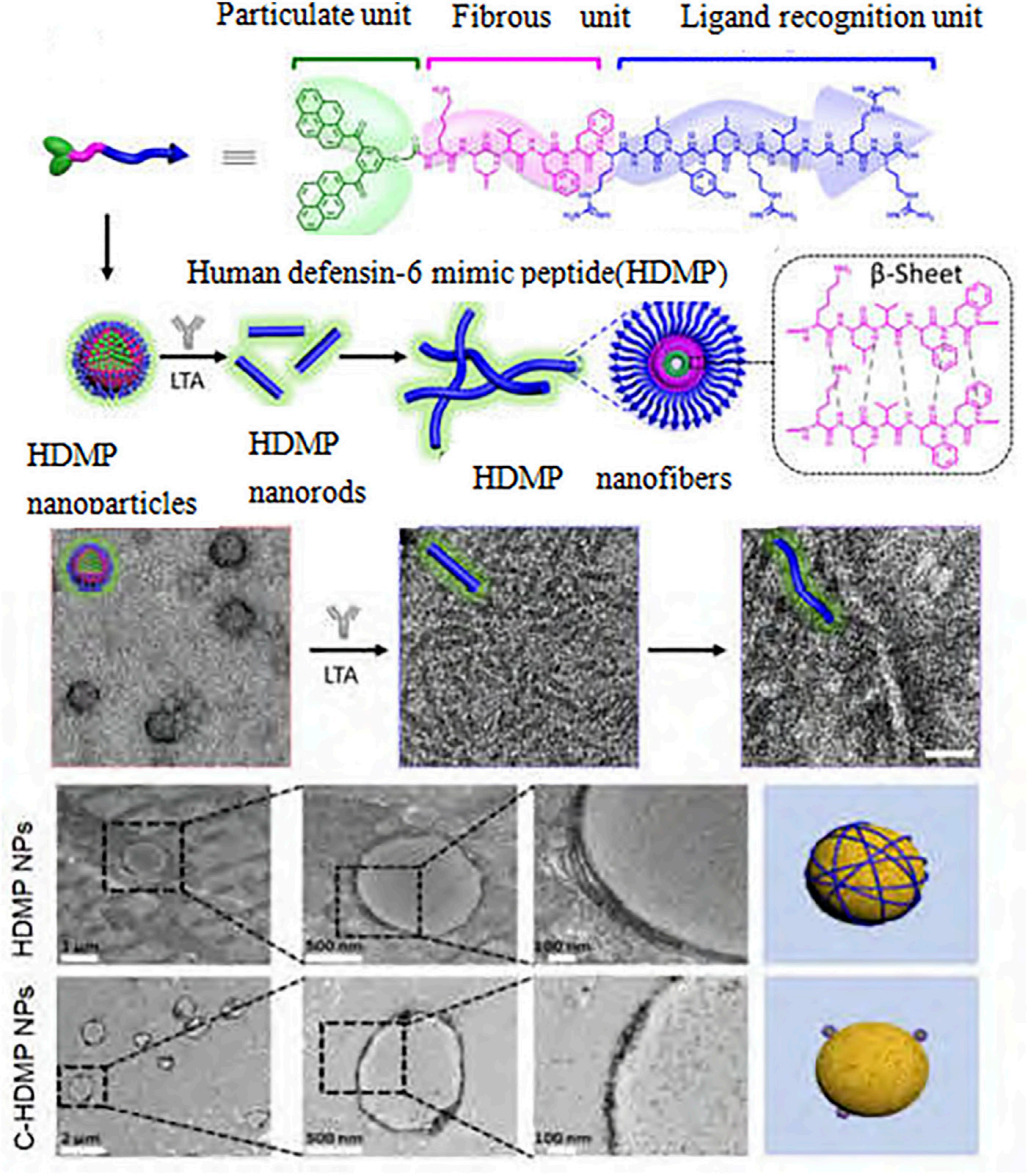
HDMP for antibacterial applications.

**FIGURE 4:**
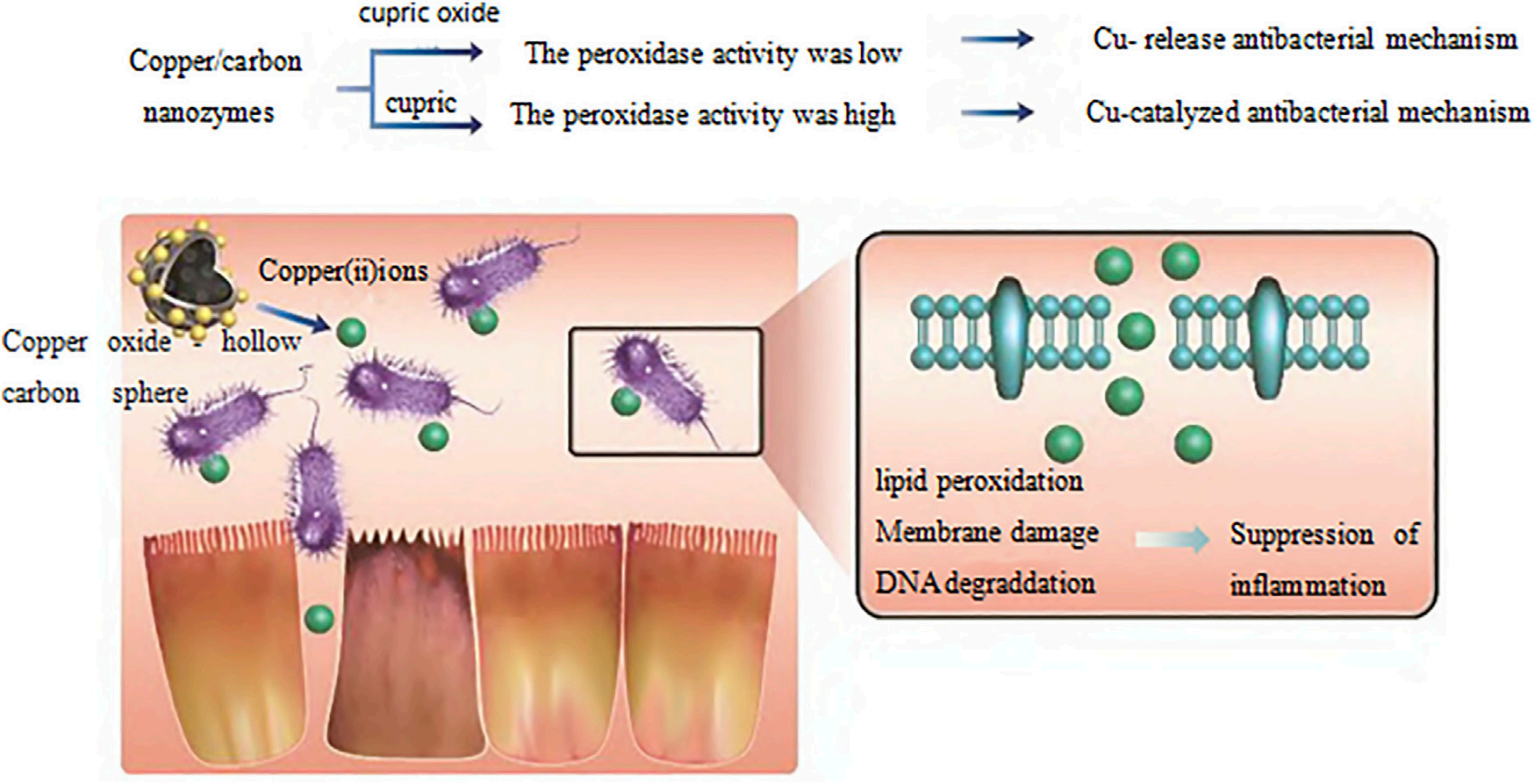
Cu/carbon hybrid nanozymes modified with different valence states.

In recent years, researchers have found that bioactive nanomaterials can play an excellent role in anti-inflammatory and anti-infection (Yang et al., 2019a). In the treatment of infectious diseases, some nanomaterials can strongly interact with cell membranes, thereby destroying the integrity of biofilms. For example, N-trimethyl chitosan nanofibers can electrostatically combine with negatively charged parts of bacterial cell wall through polycations on the membrane to generate pressure, leading to lysis and death of bacterial cells (Cheah et al., 2019). Zinc oxide nanoparticles have a positive charge, which can bind to and damage the negatively charged bacterial cell membrane, leading to the leakage of bacterial cell contents and bacterial death (Król et al., 2017). However, gold nanonail with high aspect ratio can induce bacterial dissolution through mechanical pressure, thus effectively inhibiting bacterial adhesion and bacterial biofilm formation (Elbourne et al., 2019). In addition, copper/carbon nanozymes modified by copper oxide can release Cu2+ and cause membrane damage of Gram-negative bacteria, while selenium nanoparticles can kill bacteria by disturbing membrane potential and destroying membrane structure (Cremonini et al., 2016; Huang et al., 2019). Other researchers (Joseph et al., 2016) have reported a series of quaternary phosphine and quaternary ammonium groups modified columnar aromatic hydrocarbons for antibacterial applications. Wang et al. (Guo et al., 2021b) constructed a new type of Guanidinium-modified pillar (Zhao et al., 2021) arene (GP5), which can rapidly combine with the negative electrical components on the biofilm and the phospholipid components on the bacterial membrane through a salt bridge to dissolve the bacteria, to achieve antibacterial and anti-infection effects. Some nanomaterials can also play a role in inhibiting bacteria by trapping or blocking bacteria. For example, Wang et al. (Zhang et al., 2020) designed a human defensin-6 mimic peptide, which can specifically recognize bacteria and form a nanofiber network *in situ* to trap bacteria (Figure 3). Other nanomaterials can directly kill bacteria by producing reactive oxygen species (ROS). Two types of nanozymes, peroxidase-like and oxidase-like active nanozymes, can catalyze the production of ROS (Gao et al., 2014; Fang et al., 2018a). For example, copper-modified copper/carbon nanozymes can kill bacteria by producing ROS through peroxidase-like catalysis (Figure 4); Metal-based nanomaterials such as Au, ZnO, TiO2 and graphene-based nanomaterials can also show good bactericidal effect by producing ROS (Liu et al., 2019).

### 4.2 Application of bioactive nanomaterials in inflammatory diseases

Routine physiological activities of the human body produce large amounts of free radicals, including reactive oxygen species (ROS) and reactive nitrogen species (RNS) (Mittal et al., 2014; Kwon et al., 2021), and the production and clearance of free radicals are balanced in the body through a variety of mechanisms (Closa, 2013). As one of the by-products of respiration, ROS play an important role in the occurrence of many inflammatory diseases. Inflammation can activate epithelial cells, neutrophils, and macrophages to produce a variety of inflammatory cytokines and other inflammatory mediators, which in turn impair the free radical scavenging function or reduce the expression of enzymes in the body (Jena et al., 2012; Piechota-Polanczyk and Fichna, 2014; Zhang et al., 2021a). The production and clearance of free radicals in the body cannot be balanced, leading to oxidative damage to proteins, DNA, and lipids. And further accelerate the progression of inflammation (Kawanishi et al., 2006; Zhu and Li, 2012; Vaghari-Tabari et al., 2018). Therefore, the timely removal of excessive free radicals plays a crucial role in inhibiting inflammation (Zhao et al., 2019; Zhang et al., 2021b; Weng et al., 2021). In recent years, nanomaterials have found many applications in scavenging ROS. Xie et al. (2022) reported a simple and inexpensive method to synthesize Mose2—PVP NPs with high physiological stability and biosafety levels, which mimicked the intrinsic antioxidant properties of superoxide dismutase (SOD), catalase (CAT), peroxidase (POD), and glutathione peroxidase (GPx). It can eliminate a variety of ROS (such as H2O2, OH and O2−) and RNS (such as DPPH) in mitochondria and cells, thereby improving acute pancreatitis (AP). In recent years, the incidence of inflammatory bowel disease (IBD) has gradually increased, and the pathogenesis of IBD is usually related to genetic, environmental, intestinal barrier, and immune factors (Ramos and Papadakis, 2019). With the increasing understanding of the pathogenesis of IBD, more and more new drugs and therapeutic avenues have been investigated, such as nanoparticles (Scarpignato and Pelosini, 2005), natural algae (Zhang et al., 2022), and hydrogels (Liu et al., 2021). Among them, hydrogels have become one of the most competitive materials due to their loose and porous 3D network structure and hydrophilicity. Hydrogels used to treat IBD are made of natural polymers such as chitosan (Xu et al., 2017), alginate (Cheng et al., 2022), hyaluronic acid (Liu et al., 2021), and dextran (Pitarresi et al., 2007) as well as proteins such as chondroitin sulfate (Zhang et al., 2019) and gelatin (Zhang et al., 2021c). A recent review (Ouyang et al., 2022) summarized relevant reports on the types of hydrogels used to load drugs, peptides, and proteins and immunomodulators, as well as probiotics, and found that hydrogel carriers have excellent physical and chemical properties and are well used in IBD treatment. In addition, phosphorus- based dendrimer-based molecules can inhibit the maturation of pro-inflammatory CD4^+^ T lymphocytes and dendritic cells (DC), Poly (ε-caprolactone)—β- poly (ethylenimine) (PCL—β- PEI) nanofibers can inhibit the secretion of cytokines α, tumor necrosis factor α (TNF- α) and interferon- γ (Ifn- γ) by dendritic cells and macrophages stimulated by CpG oligodeoxynucleotides (ODNs) through electrostatic adsorption. Thus, the nanofibers can inhibit inflammation (Kang and Yoo, 2014). Gold nanoparticles in precious metal nanomaterials can treat liver injury in rats by regulating AKT/PI3K and MAPK signaling pathways, downregulating the activity of Kupffer cells and hepatic stellate cells in the liver, inhibiting proinflammatory cytokines oxidative stress and fibrosis (De Carvalho et al., 2018). Zinc oxide nanoparticles in metal oxide nanomaterials can inhibit the secretion of pro-inflammatory cytokines IL-1 β and TNF- α and the activity of peroxidase in the colitis model by down-regulating the production of ROS and malondialdehyde in the colon (Li et al., 2017b). Selenium nanoparticles can protect the intestinal barrier from oxidative stress-induced inflammatory damage by activating Nrf2 and its downstream genes. Song et al. (2017) In addition, PLGA bioactive cellmionic nanoparticles coated with neutrophil membranes can effectively a dsorb the pro-inflammatory cytokines TNF- α and IL-1β in the joint cavity of RA. In addition, PLGA bioactive nanoparticles can effectively adsorb the proinflammatory factors Tnf- α and Il-1β in the joint space of RA (Deng et al., 2018).

### 4.3 Application of bioactive nanomaterials in cancer therapy

In recent years, bioactive nanomaterials have also been more and more widely used in cancer treatment. For example, PLGA bioactive cell-like nanomaterials coated with natural killer cell membranes can induce or enhance the polarization of local M1 macrophages in tumors to play an anti-tumor role (Fang et al., 2018b). Polyethylene glycol (PEG) modified polyacylthiourea tree molecules can down-regulate the expression of vascular endothelial growth factor (VEGF) at the tumor site and inhibit the formation of tumor neovascularization by highly efficient chelation of copper ions, thereby inhibiting tumor cells and tumor metastasis (Shao et al., 2017). In addition, graphite oxide acetylene (GDYO) can interact with signal transduction proteins and transcription factor STAT3 to reverse the tumor immunosuppressive microenvironment, thereby improving the role of tumor immunotherapy (Guo et al., 2021a). Gold nanoparticles can inhibit the growth of prostate cancer cells by inhibiting the expression of related metalloproteinases (Hao et al., 2021). Copper oxide and iron oxide nanomaterials can cause leakage of tumor cell membrane. Copper oxide and iron oxide nanoparticles play an anti-tumor role by activating caspase-9 and caspase-3 mediated pro-apoptotic effects (Nagajyothi et al., 2017; Yousefvand et al., 2021), while zinc oxide nanoparticles can kill tumor cells by inducing oxidative stress and pro-apoptotic pathways (Wahab et al., 2014). Black phosphorus (BP) nanosheets have shown great anticancer potential by causing cell multipolar spindle and mitosis to be delayed, and eventually cell apoptosis (Shao et al., 2021). In addition, catalase like active nanoenzymes catalyze hydrogen peroxide to generate oxygen at the tumor site, thereby enhancing the anti-tumor effect of photodynamic therapy or photothermal therapy. Bioactive nanomaterials can also regulate the interaction between Tumor-associated antigens (TAAs) and APC, thereby enhancing the uptake and presentation of TAAs by APC, thereby enhancing the degree of immune activation and playing an anti-tumor effect. For example, Min et al. (2017) constructed a biodegradable antigen-capturing nanoparticles (AC-NPs) based on Poly (lactic-co-glycolic acid) (PLGA). Yang et al. (2020) proposed a mannose-modified stearic acid-grafted chitosan micellar particle (MChSA), and (Wang et al., 2019b) proposed a Upconverting nanoparticle (UCNP) antigen-capturing nano system (UCNP/ICG/RB-MAL), thereby promoting antigen presentation and inducing tumor-specific immune response to play an anti-tumor effect. It has also been reported that nanoparticles effectively promote the maturation of APC by directly activating inflammatory cytokine receptors, thereby inducing T cell-mediated anti-tumor immunity (Roy et al., 2014). It has also been found (Kim et al., 2019) that folate-functionalized bioactive glass nanoparticles BGN (F) can effectively alleviate the immunosuppression of TME by depleting or repolarizing immunosuppressive cells. Lee et al. (2021) reported an antibody-like polymer nanoparticle (APN), which can effectively remove the immunosuppressive factor Gal-1 in the tumor, to alleviate the immunosuppression of TME and achieve the effect of anti-tumor immunity. In addition, Zhang et al. (2021d) designed a hydrogel that combines the ability of tumor photodynamic therapy (PDT) and photothermal therapy (PTT) for anti-tumor recurrence. This hydrogel is biocompatible and biodegradable, with good photothermal conversion, drug loading and CT imaging capabilities, laying the foundation for the rational design of biodegradable multifunctional hydrogels. Liu et al. (2022) reported the use of a gelatin methacrylate/oxidized dextran/montmorilite-strontium/polypyrrole (GOMP) hydrogel for synergistic treatment of osteosarcoma and potential bone regeneration. This hydrogel has a dual network structure, formed by photoinitiator-initiated double bond polymerization and Schiff base reaction. The hydrogel has good biocompatibility and excellent biodegradability *in vitro* and *in vivo*. This multifunctional DOX-loaded GOMP hydrogel with bone regeneration, photothermal therapy, and chemotherapy functions has great potential for application in the treatment of osteosarcoma.

### 4.4 Application of bioactive nanomaterials in the treatment of neurodegenerative diseases

Neurodegenerative diseases (ND), usually referring to Alzheimer’s disease, Parkinson’s disease, etc., (Marie-Therese, 2016). affect many people worldwide and are often debilitating; unfortunately, there are few treatment options for such diseases. The application of some bioactive nanomaterials with unique properties, which can play a role by inhibiting protein aggregation or eliminating formed protein aggregates, has shown great potential in the treatment of neurodegenerative diseases, providing more options for therapeutic drugs. It has been found that (Huang et al., 2014) Mixed shells polymer micelles (MSPMs) which has a unique surface phase separation structure composed of hydrophilic chain segments and hydrophobic microregions can be used for the treatment of AD. In addition, Yang et al. (2019b) reported A bioactive nanocomposite with a surface-integrated Aβ-capturing peptide (LVFF), and Xu et al. (2019) reported a new method for the treatment of AD by co-assembling Calixarene (CA) and Cyclodextrin (CD) and the preparation of supramolecular nanoparticles (CA-CD), Zhu et al. (2021) reported a TLK [(D)-TLKIVW] integrated polymer micelle particles, etc., which can play a role by inhibiting protein aggregation. In addition, in the treatment of Alzheimer’s disease, polyamide amine dendrimer molecules with carboxyl and benzene rings can inhibit the aggregation of β-amyloid peptides through hydrophobic binding and electrostatic repulsion, thus playing an anti-Alzheimer’s disease function (Wang et al., 2018; Wang et al., 2019a). It has also been found that bioactive nanomaterials can promote the removal of protein aggregates by regulating the interaction between microglia and proteins (Waisman et al., 2015; Pan et al., 2021) combined Aβ42 with A novel Aβ inhibitor (Gca-CD) to form A positively charged Gca-CD/Aβ copolymer to promote the removal of Aβ aggregates by microglia. Gu et al. (2021) reported A neuroprotective nano-scavenger that could remove Aβ oligomers from the brain and significantly improve the cognitive behavior of AD mice.

## 5 Discussion

The application of bioactive nanomaterials in biomedicine provides more options for the treatment of diseases. Compared with traditional nanomedicine preparations, bioactive nanomaterials do not exert drug effects through drug release but rely on their physical and chemical properties to interact with proteins, cells, or tissues *in vivo* and cause biological reactions to play a role in the treatment of diseases. Their biological activity is mainly affected by their physical structure, surface properties, and nanomorphology. In recent years, bioactive nanomaterials have been more and more widely studied and applied in the treatment of diseases, such as anti-inflammatory diseases, anti-infectious diseases, anti-tumor, and anti-neurodegenerative diseases. However, the development of bioactive nanomaterials still faces some challenges, such as biological effect evaluation, *in vivo* fate, structure-activity relationship, and clinical translation, etc. Further research is still needed. The main problems are summarized as follows:

(1) There are many uncertainties in the mechanism of action: At present, most of the pharmacological activities of bioactive nanomaterials refer to the ideas of pharmacological activities of small molecule drugs. However, the structure-activity relationship related to their special physical and chemical characteristics, such as size effect, interface effect, and mechanical properties, still need to be further studied to provide guidance for the rational design and development of bioactive nanomaterials.
(2) Lack of *in vivo* safety evaluation: At present, most of the research on bioactive nanomaterials focuses on their biological activity and mechanism of action, such as bioactive tree molecules, which lack safety and biocompatibility evaluation, and mostly stay at the *in vitro* level. The research on *in vivo* metabolism needs to be further improved, and the distribution, metabolism, and clearance process *in vivo* need to be further studied. In addition, the tissue targeting, biodistribution, biodegradation and immunogenicity of biomaterials need to be further solved to accelerate the *in vivo* application and clinical transformation of bioactive nanomaterials.
(3) Lack of safe and effective nanomaterials: The safety of bioactive nanomaterials includes the safety of the starting materials for preparing nanomaterials and the safety of nanomaterials themselves, and their pharmacological activity is closely related to their physical and chemical properties. Therefore, it is still a great challenge to develop nanomaterials with good biological safety. Therefore, the development of nanomaterials with good biological safety is still a great challenge. The processes and technologies that are suitable for the preparation of bioactive nanomaterials on industrial scale while ensuring uniformity and batch-to-batch stability need to be further developed.
(4) Clinical transformation needs to be further studied: The clinical application and research of bioactive nanomaterials have obvious interdisciplinary aspects, including nanoscience, materials science and engineering, and life science. In the future, it is necessary to strengthen the cooperation and communication among various disciplines, integrate advantages, and focus on the safety evaluation of materials *in vivo*, how to prepare, sterilization and storage in large amounts, to accelerate the clinical translation of bioactive nanomaterials.

Although there are still various problems in the clinical translation and application of bioactive nanomaterials, with the continuous deepening of research and breakthroughs in key scientific issues, it is believed that bioactive nanomaterials will play a greater role in the treatment of diseases in the future.

## References

[B1] Abd-El-Aziz A. S. Agatemor C. Etkin N. Overy D. P. Lanteigne M. McQuillan K. (2015). Antimicrobial organometallic dendrimers with tunable activity against multidrug-resistant bacteria. Biomacromolecules 16 (11), 3694–3703. 10.1021/acs.biomac.5b01207 26452022

[B2] Cheah W. Y. Show P. L. Ng I. S. Lin G. Y. Chiu C. Y. Chang Y. K. (2019). Antibacterial activity of quaternized chitosan modified nanofiber membrane. Int. J. Biol. Macromol. 126, 569–577. 10.1016/j.ijbiomac.2018.12.193 30584947

[B3] Chen W. Villa-Diaz L. G. Sun Y. Weng S. Kim J. K. Lam R. H. W. (2012). Nanotopography influences adhesion, spreading, and self-renewal of human embryonic stem cells. ACS Nano 6 (5), 4094–4103. 10.1021/nn3004923 22486594 PMC3358529

[B4] Cheng C. Cheng Y. Zhao S. Wang Q. Li S. Chen X. (2022). Multifunctional nanozyme hydrogel with mucosal healing activity for single-dose ulcerative colitis therapy. Bioconjug Chem. 33 (1), 248–259. 10.1021/acs.bioconjchem.1c00583 34936326

[B5] Cheng L. Wang X. Gong F. Liu T. Liu Z. (2020). 2D nanomaterials for cancer theranostic applications. Adv. Mater 32 (13), e1902333. 10.1002/adma.201902333 31353752

[B6] Cheng Y. Xiao X. Li X. Song D. Lu Z. Wang F. (2017). Characterization, antioxidant property and cytoprotection of exopolysaccharide-capped elemental selenium particles synthesized by Bacillus paralicheniformis SR14. Carbohydr. Polym. 178, 18–26. 10.1016/j.carbpol.2017.08.124 29050583

[B7] Closa D. (2013). Free radicals and acute pancreatitis: Much ado about … something something. Free Radic. Res. 47, 934–940. 10.3109/10715762.2013.829571 23895210

[B8] Cremonini E. Zonaro E. Donini M. Lampis S. Boaretti M. Dusi S. (2016). Biogenic selenium nanoparticles: Characterization, antimicrobial activity and effects on human dendritic cells and fibroblasts. Microb. Biotechnol. 9 (6), 758–771. 10.1111/1751-7915.12374 27319803 PMC5072192

[B9] De Carvalho T. G. Garcia V. B. De Araújo A. A. da Silva Gasparotto L. H. Silva H. Guerra G. C. B. (2018). Spherical neutral gold nanoparticles improve anti-inflammatory response, oxidative stress and fibrosis in alcohol-methamphetamine-induced liver injury in rats. Int. J. Pharm. 548 (1), 1–14. 10.1016/j.ijpharm.2018.06.008 29886101

[B10] Deng G. Sun Z. Li S. Peng X. Li W. Zhou L. (2018). Cell-membrane immunotherapy based on natural killer cell membrane coated nanoparticles for the effective inhibition of primary and abscopal tumor growth. ACS Nano 12 (12), 12096–12108. 10.1021/acsnano.8b05292 30444351

[B11] Dong Y. Li W. Gu Z. Xing R. Ma Y. Zhang Q. (2019). Inhibition of HER2-positive breast cancer growth by blocking the HER2 signaling pathway with HER2-glycan-imprinted nanoparticles. Angew. Chem. Int. Ed. Engl. 58 (31), 10621–10625. 10.1002/anie.201904860 31166063

[B12] Eivazzadeh-Keihan R. Bahojb Noruzi E. Khanmohammadi Chenab K. Jafari A. Radinekiyan F. Hashemi S. M. (2020). Metal-based nanoparticles for bone tissue engineering. J. Tissue Eng. Regen. Med. 14 (12), 1687–1714. 10.1002/term.3131 32914573

[B13] Elbourne A. Coyle V. E. Truong V. K. Sabri Y. M. Kandjani A. E. Bhargava S. K. (2019). Multi-directional electrodeposited gold nanospikes for antibacterial surface applications. Nanoscale Adv. 1 (1), 203–212. 10.1039/c8na00124c 36132449 PMC9473181

[B14] Fan K. Xi J. Fan L. Wang P. Zhu C. Tang Y. (2018). *In vivo* guiding nitrogen-doped carbon nanozyme for tumor catalytic therapy. Nat. Commun. 9 (1), 1440. 10.1038/s41467-018-03903-8 29650959 PMC5897348

[B15] Fang G. Li W. Shen X. Perez-Aguilar J. M. Chong Y. Gao X. (2018). Differential Pd-nanocrystal facets demonstrate distinct antibacterial activity against Gram-positive and Gram-negative bacteria. Nat. Commun. 9 (1), 129. 10.1038/s41467-017-02502-3 29317632 PMC5760645

[B16] Fang R. H. Kroll A. V. Gao W. Zhang L. (2018). Cell membrane coating nanotechnology. J. Adv. Mater. 30 (23), 1706759. 10.1002/adma.201706759 PMC5984176 29582476

[B17] Gao L. Giglio K. M. Nelson J. L. Sondermann H. Travis A. J. (2014). Ferromagnetic nanoparticles with peroxidase-like activity enhance the cleavage of biological macromolecules for biofilm elimination. Nanoscale 6 (5), 2588–2593. 10.1039/c3nr05422e 24468900 PMC3951791

[B18] Gu Y. Zhao Y. Zhang Z. Hao J. Zheng Y. Liu Q. (2021). An antibody-like polymeric nanoparticle removes intratumoral galectin-1 to enhance antitumor T-cell responses in cancer immunotherapy. ACS Appl. Mater Interfaces 13 (19), 22159–22168. 10.1021/acsami.1c02116 33955217

[B19] Guo S. Zhao L. Liu J. Wang X. Yao H. Chang X. (2021a). The underlying function and structural organization of the intracellular protein corona on graphdiyne oxide nanosheet for local immunomodulation. Nano Lett. 21 (14), 6005–6013. 10.1021/acs.nanolett.1c01048 34242035

[B20] Guo S. Huang Q. Chen Y. Wei J. Zheng J. Wang L. (2021b). Synthesis and bioactivity of guanidinium-functionalized pillar [5] arene as a biofilm disruptor. Angew. Chem. Int. Ed. Engl. 60 (2), 618–623. 10.1002/anie.202013975 33128291

[B21] Hao Y. Hu J. Wang H. Wang C. (2021). Gold nanoparticles regulate the antitumor secretome and have potent cytotoxic effects against prostate cancer cells. J. Appl. Toxicol. 41 (8), 1286–1303. 10.1002/jat.4117 33355407

[B22] Henchll P. J. M. (2002). Third-generation biomedicalmaterials. Science 295 (5557), 1014–1017. 10.1126/science.1067404 11834817

[B23] Huang F. Wang J. Qu A. Shen L. Liu J. Liu J. (2014). Maintenance of amyloid β peptide homeostasis by artificial chaperones based on mixed-shell polymeric micelles. Angew. Chem. Int. Ed. Engl. 53 (34), 8985–8990. 10.1002/anie.201400735 24985739

[B24] Huang T. Holden J. A. Heath D. E. O'Brien-Simpson N. M. O'Connor A. J. (2019). Engineering highly effective antimicrobial selenium nanoparticles through control of particle size. Nanoscale 11 (31), 14937–14951. 10.1039/c9nr04424h 31363721

[B25] Huang Z. Liu Y. Wang L. Ali A. Yao Q. Jiang X. (2020). Supramolecular assemblies mimicking neutrophil extracellular traps for MRSE infection control. biomaterials 253, 120124. 10.1016/j.biomaterials.2020.120124 32447104

[B26] Islam M. M. Shahruzzaman M. Biswas S. Nurus Sakib M. Rashid T. U. (2020). Chitosan based bioactive materials in tissue engineering applications-A review. Bioact. Mater 5 (1), 164–183. 10.1016/j.bioactmat.2020.01.012 32083230 PMC7016353

[B27] Jena G. Trivedi P. P. Sandala B. (2012). Oxidative stress in ulcerative colitis: An old concept but a new concern. Free Radic. Res. 46, 1339–1345. 10.3109/10715762.2012.717692 22856328

[B28] Joseph R. Kaizerman D. Herzog I. M. Hadar M. Feldman M. Fridman M. (2016). Phosphonium pillar [5] arenes as a new class of efficient biofilm inhibitors: Importance of charge cooperativity and the pillar platform. Chem. Commun. (Camb) 52 (70), 10656–10659. 10.1039/c6cc05170g 27503150

[B29] Kang J. Yoo H. S. (2014). Nucleic acid-scavenging electrospun nanofibrous meshes for suppressing inflammatory responses. Biomacromolecules 15 (7), 2600–2606. 10.1021/bm500437e 24884211

[B30] Kawanishi S. Hiraku Y. Pinlaor S. (2006). Oxidative and nitrative DNA damage in animals and patients with inflammatory diseases in relation to inflammation-related carcinogenesis. Biol. Chem. 387, 365–372. 10.1515/BC.2006.049 16606333

[B31] Kerativitayanan P. Carrow J. K. Gaharwar A. K. (2015). Nanomaterials for engineering stem cell responses. Adv. Healthc. Mater 4 (11), 1600–1627. 10.1002/adhm.201500272 26010739

[B32] Kim T. H. Kang M. S. Mandakhbayar N. El-Fiqi A. Kim H. W. (2019). Anti-inflammatory actions of folate-functionalized bioactive ion-releasing nanoparticles imply drug-free nanotherapy of inflamed tissues. Biomaterials 207, 23–38. 10.1016/j.biomaterials.2019.03.034 30952042

[B33] Koide H. Yoshimatsu K. Hoshino Y. Lee S. H. Okajima A. Ariizumi S. (2017). A polymer nanoparticle with engineered affinity for a vascular endothelial growth factor (VEGF165). Nat. Chem. 9 (7), 715–722. 10.1038/nchem.2749 28644480

[B34] Król A. Pomastowski P. Rafińska K. Railean-Plugaru V. Buszewski B. (2017). Zinc oxide nanoparticles: Synthesis, antiseptic activity and toxicity mechanism. Adv. Colloid Interface Sci. 249, 37–52. 10.1016/j.cis.2017.07.033 28923702

[B35] Kumawat M. K. Thakur M. Gurung R. B. Srivastava R. (2017). Graphene quantum dots for cell proliferation, nucleus imaging, and photoluminescent sensing applications. Sci. Rep. 7 (1), 15858. 10.1038/s41598-017-16025-w 29158566 PMC5696518

[B36] Kwon K. W. Park H. Song K. H. Choi J. C. Ahn H. Park M. J. (2012). Nanotopography-guided migration of T cells. J. Immunol. 189 (5), 2266–2273. 10.4049/jimmunol.1102273 22844118

[B37] Kwon N. Kim D. Swamy K. M. K. Yoon J. (2021). Metal-coordinated fluorescent and luminescent probes for reactive oxygen species (ROS) and reactive nitrogen species (RNS). Coord. Chem. Rev. 427, 213581. 10.1016/j.ccr.2020.213581

[B38] Lee J. H. Shin G. Baek J. Y. Kang T. J. (2021). An electricity-generating window made of aTransparent energy harvester of thermocells. ACS Appl. Mater Interfaces 13 (18), 21157–21165. 10.1021/acsami.1c00164 33793183

[B39] Li S. Chen H. Wang B. Cai C. Yang X. Chai Z. (2017b). ZnO nanoparticles act as supportive therapy in DSS-induced ulcerative colitis in mice by maintaining gut homeostasis and activating Nrf2 signaling. Sci. Rep. 7 (1), 43126. 10.1038/srep43126 28233796 PMC5324050

[B40] Li S. Zhou S. Li Y. Li X. Zhu J. Fan L. (2017a). Exceptionally high payload of the IR780 iodide on folic acid-functionalized graphene quantum dots for targeted photothermal therapy. ACS Appl. Mater Interfaces 9 (27), 22332–22341. 10.1021/acsami.7b07267 28643511

[B41] Lin G. Mi P. Chu C. Zhang J. Liu G. (2016). Inorganic nanocarriers overcoming multidrug resistance for cancer theranostics. Adv. Sci. (Weinh). 3 (11), 1600134. 10.1002/advs.201600134 27980988 PMC5102675

[B42] Liu H. Cai Z. Wang F. Hong L. Deng L. Zhong J. (2021). Colon-targeted adhesive hydrogel microsphere for regulation of gut immunity and flora. Adv. Sci. (Weinh). 8 (18), e2101619. 10.1002/advs.202101619 34292669 PMC8456273

[B43] Liu X. Zhang Y. Wu H. Tang J. Zhou J. Zhao J. (2022). A conductive gelatin methacrylamide hydrogel for synergistic therapy of osteosarcoma and potential bone regeneration. Int. J. Biol. Macromol. 228, 111–122. 10.1016/j.ijbiomac.2022.12.185 36563819

[B44] Liu Y. ShiSu L. L. van der Mei H. C. Jutte P. C. Ren Y. (2019). Nanotechnology-based antimicrobials and delivery systems for biofilm-infection control. Chem. Soc. Rev. 48 (2), 428–446. 10.1039/c7cs00807d 30601473

[B45] Marie-Therese S. (2016). Neurodegenerative diseases [J]. Nat. Heemels 539 (7628), 179–180. 10.1038/539179a 27830810

[B46] Michaud G. Visini R. Bergmann M. Salerno G. Bosco R. Gillon E. (2016). Overcoming antibiotic resistance in *Pseudomonas aeruginosa* biofilms using glycopeptide dendrimers. Chem. Sci. 7 (1), 166–182. 10.1039/c5sc03635f 29896342 PMC5953009

[B47] Min Y. Roche K. C. Tian S. Eblan M. J. McKinnon K. P. Caster J. M. (2017). Antigen-capturing nanoparticles improve the abscopal effect and cancer immunotherapy. Nat. Nanotechnol. 12 (9), 877–882. 10.1038/nnano.2017.113 28650437 PMC5587366

[B48] Mintzer M. A. Dane E. L. O’toole G. A. Grinstaff M. W. (2012). Exploiting dendrimer multivalency to combat emerging and re-emerging infectious diseases. Mol. Pharm. 9 (3), 342–354. 10.1021/mp2005033 22126461 PMC3729585

[B49] Mittal M. Siddiqui M. R. Tran K. Reddy S. P. Malik A. B. (2014). Reactive oxygen species in inflammation and tissue injury. Antioxid. Redox Signal 20, 1126–1167. 10.1089/ars.2012.5149 23991888 PMC3929010

[B50] Nagajyothi P. C. Pandurangan M. Kim D. H. Sreekanth T. V. M. Shim J. (2017). Green synthesis of iron oxide nanoparticles and their catalytic and *in vitro* anticancer activities. J. Clust. Sci. 28 (1), 245–257. 10.1007/s10876-016-1082-z

[B51] Nethi S. K. Das S. Patra C. R. Mukherjee S. (2019). Recent advances in inorganic nanomaterials for wound-healing applications. Biomater. Sci. 7 (7), 2652–2674. 10.1039/c9bm00423h 31094374

[B52] Ouyang Y. Zhao J. Wang S. (2022). Multifunctional hydrogels based on chitosan, hyaluronic acid and other biological macromolecules for the treatment of inflammatory bowel disease: A review. Int. J. Biol. Macromol. 227, 505–523. 10.1016/j.ijbiomac.2022.12.032 36495992

[B53] Pan Y. Wang H. Xu X. (2021). Coassembly of macrocyclic amphiphiles for anti-β-amyloid therapy of Alzheimer’s disease [J]. CCS Chem. 3 (9), 2485–2497.

[B54] Park M. V. Neigh A. M. Vermeulen J. P. de la Fonteyne L. J. J. Verharen H. W. Briede J. J. (2011). The effect of particle size on the cytotoxicity, inflammation, developmental toxicity, and genotoxicity of silver nanoparticles. Biomaterials 32 (36), 9810–9817. 10.1016/j.biomaterials.2011.08.085 21944826

[B55] Piechota-Polanczyk A. Fichna J. (2014). Review article: The role of oxidative stress in pathogenesis and treatment of inflammatory bowel diseases. Schmiedeb. Arch. Pharmacol. 387, 605–620. 10.1007/s00210-014-0985-1 PMC4065336 24798211

[B56] Pitarresi G. Casadei M. A. Mandracchia D. Paolicelli P. Palumbo F. S. Giammona G. (2007). Photocrosslinking of dextran and polyaspartamide derivatives: A combination suitable for colon-specific drug delivery. J. Control Release 119 (3), 328–338. 10.1016/j.jconrel.2007.03.005 17475357

[B57] Ramos G. P. Papadakis K. A. (2019). Mechanisms of disease: Inflammatory bowel diseases. Mayo Clin. Proc. 94 (1), 155–165. 10.1016/j.mayocp.2018.09.013 30611442 PMC6386158

[B58] Roy R. Singh S. K. Das M. Tripathi A. Dwivedi P. D. (2014). Toll-like receptor 6 mediated inflammatory and functional responses of zinc oxide nanoparticles Primed macrophages. Immunology 142 (3), 453–464. 10.1111/imm.12276 24593842 PMC4080961

[B59] Scarpignato C. Pelosini I. (2005). Rifaximin, a poorly absorbed antibiotic: Pharmacology and clinical potential. Chemotherapy 51 (1), 36–66. 10.1159/000081990 15855748

[B60] Seisenbaeva G. A. Fromell K. Vinogradov V. V. Terekhov A. N. Pakhomov A. V. Nilsson B. (2017). Dispersion of TiO_2_ nanoparticles improves burn wound healing and tissue regeneration through specific interaction with blood serum proteins [J]. Sci. Rep. 7 (1), 15448. 10.1038/s41598-017-15792-w 29133853 PMC5684224

[B61] Shao S. Zhou Q. Si J. Tang J. Liu X. Wang M. (2017). A non-cytotoxic dendrimer with innate and potent anticancer and anti-metastatic activities. Nat. Biomed. Eng. 1 (9), 745–757. 10.1038/s41551-017-0130-9 31015667

[B62] Shao X. Ding Z. Zhou W. Li Y. Cui H. (2021). Intrinsic bioactivity of black phosphorus nanomaterials on mitotic centrosome destabilization through suppression of PLK1 kinase. Nat. Nanotechnol. 16, 1150–1160. 10.1038/s41565-021-00952-x 34354264

[B63] Shikhi-Abadi P. G. Irani M. (2021). A review on the applications of electrospun chitosan nanofibers for the cancer treatment. Int. J. Biol. Macromol. 183, 790–810. 10.1016/j.ijbiomac.2021.05.009 33965480

[B64] Song D. Cheng Y. Li X. Wang F. Lu Z. Xiao X. (2017). Biogenic nanoselenium particles effectively attenuate oxidative stress-induced intestinal epithelial barrier injury by activating the Nrf2 antioxidant pathway. ACS Appl. Mater. interfaces 9 (17), 14724–14740. 10.1021/acsami.7b03377 28406025

[B65] Tang X. Li F. Jia J. Yang C. Liu W. Jin B. (2017). Synthesis of magnetic molecularly imprinted polymers with excellent biocompatibility for the selective separation and inhibition of testosterone in prostate cancer cells. Int. J. Nanomedicine 12, 2979–2993. 10.2147/IJN.S133009 28442907 PMC5396939

[B66] Tiwari J. N. Vij V. Kemp K. C. Kim K. S. (2016). Engineered carbon-nanomaterial-based electrochemical sensors for biomolecules. ACS Nano 10 (1), 46–80. 10.1021/acsnano.5b05690 26579616

[B67] Vaghari-Tabari M. Moein S. Qujeq D. Kashifard M. Hajian-Tilaki K. (2018). Positive correlation of fecal calprotectin with serum antioxidant enzymes in patients with inflammatory bowel disease: Accidental numerical correlation or a new finding? Am. J. Med. Sci. 355, 449–455. 10.1016/j.amjms.2017.12.009 29753375

[B68] Wahab R. Siddiqui M. A. Saquib Q. Dwivedi S. Ahmad J. Musarrat J. (2014). ZnO nanoparticles induced oxidative stress and apoptosis in HepG2 and MCF-7 cancer cells and their antibacterial activity. Colloids Surfaces B Biointerfaces 117, 267–276. 10.1016/j.colsurfb.2014.02.038 24657613

[B69] Waisman A. Liblau R. S. Becher B. (2015). Innate and adaptive immune responses in the CNS. Lancet Neurol. 14 (9), 945–955. 10.1016/S1474-4422(15)00141-6 26293566

[B70] Wang L. Wang X. Bhirde A. Cao J. Zeng Y. Huang X. (2014). Carbon-dot-based two-photon visible nanocarriers for safe and highly efficient delivery of siRNA and DNA. Adv. Healthc. Mater 3 (8), 1203–1209. 10.1002/adhm.201300611 24692418 PMC4134771

[B71] Wang Z. Song J. Zhou F. Hoover A. R. Murray C. Zhou B. (2019b). NIR-triggered phototherapy, and immunotherapy via an antigen-capturing nanoplatform for metastatic cancer treatment. Adv. Sci. (Weinh) 6 (10), 1802157. 10.1002/advs.201802157 31131193 PMC6523374

[B72] Wang X. Hu Y. Wei H. (2016). Nanozymes in bionanotechnology: From sensing to therapeutics and beyond. Inorg. Chem. Front. 3 (1), 41–60. 10.1039/c5qi00240k

[B73] Wang Z. Dong X. Sun Y. (2018). Hydrophobic modification of carboxyl-terminated polyamidoamine dendrimer surface creates a potent inhibitor of amyloid-β fibrillation. Langmuir 34 (47), 14419–14427. 10.1021/acs.langmuir.8b02890 30388015

[B74] Wang Z. Dong X. Sun Y. (2019a). Mixed carboxyl and hydrophobic dendrimer surface inhibits amyloid-β fibrillation: New insight from the generation number effect. Langmuir 35 (45), 14681–14687. 10.1021/acs.langmuir.9b02527 31635460

[B75] Wei H. Wang E. (2013). Nanomaterials with enzyme-like characteristics (nanozymes): Next-generation artificial enzymes. Chem. Soc. Rev. 42 (14), 6060–6093. 10.1039/c3cs35486e 23740388

[B76] Weng Q. J. Sun H. Fang C. Y. Xia F. Liao H. Lee J. (2021). Catalytic activity tunable ceria nanoparticles prevent chemotherapy-induced acute kidney injury without interference with chemotherapeutics. Nat. Commun. 12, 1436. 10.1038/s41467-021-21714-2 33664241 PMC7933428

[B77] Wu J. Wang X. Wang Q. Lou Z. Li S. Zhu Y. (2019). Nanomaterials with enzyme-like characteristics (nanozymes): Next-generation artificial enzymes (II). Chem. Soc. Rev. 48 (4), 1004–1076. 10.1039/c8cs00457a 30534770

[B78] Wu Y. Fan Q. Zeng F. Zhu J. Chen J. Fan D. (2018). Peptide-functionalized nanoinhibitor restrains brain tumor growth by abrogating mesenchymal-epithelial transition factor (MET) signaling. Nano Lett. 18 (9), 5488–5498. 10.1021/acs.nanolett.8b01879 30067910

[B79] Xie P. Zhang L. Shen H. Wu H. Zhao J. Wang S. (2022). Biodegradable MoSe2-polyvinylpyrrolidone nanoparticles with multi-enzyme activity for ameliorating acute pancreatitis. J. Nanobiotechnology 20 (1), 113. 10.1186/s12951-022-01288-x 35248068 PMC8898412

[B80] Xiong Z. Zhang X. Zhang S. Lei L. Ma W. Li D. (2018). Bacterial toxicity of exfoliated black phosphorus nanosheets. Ecotoxicol. Environ. Saf. 161, 507–514. 10.1016/j.ecoenv.2018.06.008 29913419

[B81] Xu J. Tam M. Samaei S. Lerouge S. Barralet J. Stevenson M. M. (2017). Mucoadhesive chitosan hydrogels as rectal drug delivery vessels to treat ulcerative colitis. Acta Biomater. 48, 247–257. 10.1016/j.actbio.2016.10.026 27769943

[B82] Xu S. Chang L. Hu Y. Zhao X. Huang S. Chen Z. (2021). Tea polyphenol modified, photothermal responsive and ROS generative black phosphorus quantum dots as nanoplatforms for promoting MRSA infected wounds healing in diabetic rats. J. Nanobiotechnology 19 (1), 362. 10.1186/s12951-021-01106-w 34758829 PMC8579683

[B83] Xu Z. Jia S. Wang W. Yuan Z. Jan Ravoo B. Guo D. S. (2019). Heteromultivalent peptide recognition by co-assembly of cyclodextrin and calixarene amphiphiles enables inhibition of amyloid fibrillation. Nat. Chem. 11 (1), 86–93. 10.1038/s41557-018-0164-y 30455432

[B84] Yang C. Lou W. Zhong G. Lee A. Leong J. Chin W. (2019a). Degradable antimicrobial polycarbonates with unexpected activity and selectivity for treating multidrug-resistant *Klebsiella pneumoniae* lung infection in mice. Acta Biomater. 94, 268–280. 10.1016/j.actbio.2019.05.057 31129359

[B85] Yang C. Li X. Zhu L. Wu X. Zhang S. Huang F. (2019b). Heat shock protein inspired nanochaperones restore amyloid-β homeostasis for preventative therapy of Alzheimer's disease. Adv. Sci. 6 (22), 1901844. 10.1002/advs.201901844 PMC6864524 31763156

[B86] Yang X. Yu T. Zeng Y. Lian K. Zhou X. Li S. (2020). Tumor-draining lymph node targeting chitosan micelles as antigen-capturing adjuvants for personalized immunotherapy. Carbohydr. Polym. 240, 116270. 10.1016/j.carbpol.2020.116270 32475559

[B87] Yousefvand P. Mohammadi E. Zhuang Y. Bloukh S. H. Edis Z. Gamasaee N. A. (2021). Biothermodynamic, antiproliferative and antimicrobial properties of synthesized copper oxide nanoparticles. J. Mol. Liq. 324, 114693. 10.1016/j.molliq.2020.114693

[B88] Zhang D. Zhong D. Ouyang J. He J. Qi Y. Chen W. (2022). Microalgae-based oral microcarriers for gut microbiota homeostasis and intestinal protection in cancer radiotherapy. Nat. Commun. 13 (1), 1413. 10.1038/s41467-022-28744-4 35301299 PMC8931093

[B89] Zhang D. Y. Liu H. K. Zhu K. S. Younis M. R. Yang C. (2021b). Prussian blue-based theranostics for ameliorating acute kidney injury. J. Nanobiotechnol 19, 266. 10.1186/s12951-021-01006-z PMC8419910 34488789

[B90] Zhang D. Y. Younis M. R. Liu H. K. Lei S. Wan Y. Qu J. (2021a). Multi-enzyme mimetic ultrasmall iridium nanozymes as reactive oxygen/nitrogen species scavengers for acute kidney injury management. Biomaterials 271, 120706. 10.1016/j.biomaterials.2021.120706 33607543

[B91] Zhang D. Y. Zheng Y. Tan C. P. Sun J. H. Zhang W. Ji L. N. (2017). Graphene oxide decorated with Ru(II)-Polyethylene glycol complex for lysosome-targeted imaging and photodynamic/photothermal therapy. ACS Appl. Mater Interfaces 9 (8), 6761–6771. 10.1021/acsami.6b13808 28150943

[B92] Zhang H. (2020). Molecularly imprinted nanoparticles for biomedical applications. Adv. Mater 32 (3), e1806328. 10.1002/adma.201806328 31090976

[B93] Zhang L. Jing D. Jiang N. Rojalin T. Baehr C. M. Zhang D. (2020). Transformable peptide nanoparticles arrest HER2 signalling and cause cancer cell death *in vivo* . Nat. Nanotechnol. 15 (2), 145–153. 10.1038/s41565-019-0626-4 31988501 PMC7147967

[B94] Zhang D. Y. Kang L. Hu S. Hu J. Fu Y. Hu Y. (2021c). Carboxymethyl chitosan microspheres loaded hyaluronic acid/gelatin hydrogels for controlled drug delivery and the treatment of inflammatory bowel disease. Int. J. Biol. Macromol. 167, 1598–1612. 10.1016/j.ijbiomac.2020.11.117 33220374

[B95] Zhang X. Ma Y. Ma L. Zu M. Song H. Xiao B. (2019). Oral administration of chondroitin sulfate-functionalized nanoparticles for colonic macrophage-targeted drug delivery. Carbohydr. Polym. 223, 115126. 10.1016/j.carbpol.2019.115126 31426992

[B96] Zhang D. Y. Zhu C. Zhang Z. Zhao J. Yuan Y. Wang S. (2021d). Oxidation triggered formation of polydopamine-modified carboxymethyl cellulose hydrogel for anti-recurrence of tumor. Colloids Surf. B Biointerfaces 207, 112025. 10.1016/j.colsurfb.2021.112025 34403982

[B97] Zhao C. Wu L. Wang X. Weng S. Ruan Z. Liu Q. (2020). Quaternary ammonium carbon quantum dots as an antimicrobial agent against gram-positive bacteria for the treatment of MRSA-infected pneumonia in mice. Carbon 163, 70–84. 10.1016/j.carbon.2020.03.009

[B98] Zhao J. L. Gao W. Cai X. J. Xu J. Zou D. Li Z. (2019). Nanozyme-mediated catalytic nanotherapy for inflammatory bowel disease. Theranostics 9, 2843–2855. 10.7150/thno.33727 31244927 PMC6568174

[B99] Zhao Y. Li Q. Chai J. Liu Y. (2021). Cargo-templated crosslinked polymer nanocapsules and their biomedical applications. Adv. NanoBiomed Res. 1 (4), 2000078. 10.1002/anbr.202000078

[B100] Zheng K. Setyawati M. I. Leong D. T. Xie J. (2017). Antimicrobial gold nanoclusters. ACS Nano 11 (7), 6904–6910. 10.1021/acsnano.7b02035 28595000

[B101] Zheng X. T. Ananthanarayanan A. Luo K. Q. Chen P. (2015). Glowing graphene quantum dots and carbon dots: Properties, syntheses, and biological applications. Small 11 (14), 1620–1636. 10.1002/smll.201402648 25521301

[B102] Zhou Q. Gu H. Sun S. Zhang Y. Hou Y. Li C. (2021). Large-sized graphene oxide nanosheets increase DC-T-cell synaptic contact and the efficacy of DC vaccines against SARS-CoV-2. Adv. Mater. 33 (40), 2102528. 10.1002/adma.202102528 34396603 PMC8420123

[B103] Zhou W. Pan T. Cui H. Zhao Z. Chu P. K. Yu X. F. (2019b). Black phosphorus: Bioactive nanomaterials with inherent and selective chemotherapeutic effects. Angew. Chem. Int. Ed. Engl. 58 (3), 769–774. 10.1002/anie.201810878 30444063

[B104] Zhou W. Pan T. Cui H. (2019a). Black phosphorus: Bioactive nanomaterials with inherent and selective chemotherapeutic effects [J]. Angew. Chem. Int. Ed. 131 (3), 779–784. 10.1002/anie.201810878 30444063

[B105] Zhu H. Li Y. R. (2012). Oxidative stress and redox signaling mechanisms of inflammatory bowel disease: Updated experimental and clinical evidence. Exp. Biol. Med. 237, 474–480. 10.1258/ebm.2011.011358 22442342

[B106] Zhu L. Xu L. Wu X. Deng F. Ma R. Liu Y. (2021). Tau-targeted multifunctional nanoinhibitor for Alzheimer's disease. ACS Appl. Mater Interfaces 13 (20), 23328–23338. 10.1021/acsami.1c00257 33999598

[B107] Zielińska P. Staniszewska M. Bondaryk M. Koronkiewicz M. Urbanczyk-Lipkowska Z. (2015). Design and studies of multiple mechanism of anti-Candida activity of a new potent Trp-rich peptide dendrimers. Eur. J. Med. Chem. 105, 106–119. 10.1016/j.ejmech.2015.10.013 26479030

